# Phylogenetic relationships of the nematode subfamily Phascolostrongylinae from macropodid and vombatid marsupials inferred using mitochondrial protein sequence data

**DOI:** 10.1186/s13071-021-05028-2

**Published:** 2021-10-09

**Authors:** Tanapan Sukee, Ian Beveridge, Anson V. Koehler, Ross Hall, Robin B. Gasser, Abdul Jabbar

**Affiliations:** 1 Department of Veterinary Biosciences, Melbourne Veterinary School, The University of Melbourne, Victoria, Australia; 2 South Australian Museum, Adelaide, South Australia Australia

**Keywords:** Phascolostrongylinae, Macropodid marsupials, Vombatid marsupials, Mitochondrial DNA

## Abstract

**Background:**

The subfamily Phascolostrongylinae (Superfamily Strongyloidea) comprises nematodes that are parasitic in the gastrointestinal tracts of macropodid (Family Macropodidae) and vombatid (Family Vombatidae) marsupials. Currently, nine genera and 20 species have been attributed to the subfamily Phascolostrongylinae. Previous studies using sequence data sets for the internal transcribed spacers (ITS) of nuclear ribosomal DNA showed conflicting topologies between the Phascolostrongylinae and related subfamilies. Therefore, the aim of this study was to validate the phylogenetic relationships within the Phascolostrongylinae and its relationship with the families Chabertiidae and Strongylidae using mitochondrial amino acid sequences.

**Methods:**

The sequences of all 12 mitochondrial protein-coding genes were obtained by next-generation sequencing of individual adult nematodes (*n* = 8) representing members of the Phascolostrongylinae. These sequences were conceptually translated and the phylogenetic relationships within the Phascolostrongylinae and its relationship with the families Chabertiidae and Strongylidae were inferred from aligned, concatenated amino acid sequence data sets.

**Results:**

Within the Phascolostrongylinae, the wombat-specific genera grouped separately from the genera occurring in macropods. Two of the phascolostrongyline tribes were monophyletic, including Phascolostrongylinea and Hypodontinea, whereas the tribe Macropostrongyloidinea was paraphyletic. The tribe Phascolostrongylinea occurring in wombats was closely related to *Oesophagostomum* spp., also from the family Chabertiidae, which formed a sister relationship with the Phascolostrongylinae.

**Conclusion:**

The current phylogenetic relationship within the subfamily Phascolostrongylinae supports findings from a previous study based on ITS sequence data. This study contributes also to the understanding of the phylogenetic position of the subfamily Phascolostrongylinae within the Chabertiidae. Future studies investigating the relationships between the Phascolostrongylinae and Cloacininae from macropodid marsupials may advance our knowledge of the phylogeny of strongyloid nematodes in marsupials.

**Graphical Abstract:**

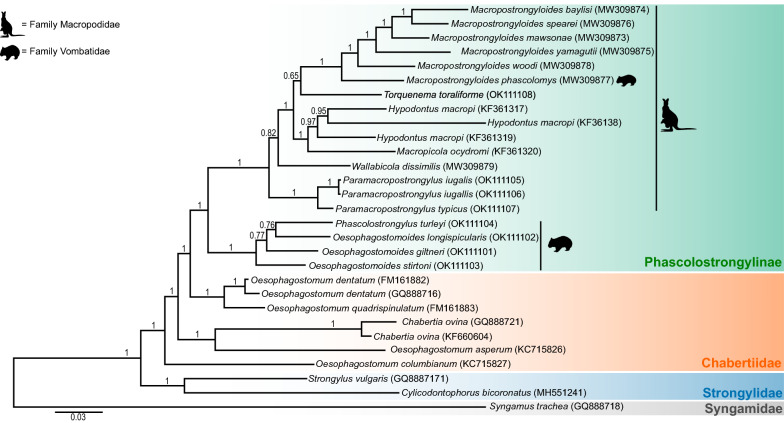

## Background

The Phascolostrongylinae is a subfamily of strongyloid nematode (Nematoda: Strongyloidea) belonging to the family Chabertiidae. The subfamily comprising nine genera and 20 species, parasitises macropodid (Family Macropodidae) and vombatid (Family Vombatidae) marsupials. Most of the phascolostrongyline genera occur within the intestines of their hosts. However, a few exceptions are found in the stomachs of their hosts, including *Paramacropostrongylus* from grey kangaroos (*Macropus* spp.) and *Wallabicola* from swamp wallabies (*Wallabia bicolor*) [1].

Genera of the Phascolostrongylinae are currently subdivided into three tribes (Phascolostrongylinea, Hypodontinea and Macropostrongyloidinea) based primarily on the features of the buccal capsule [2]. The tribe Phascolostrongylinea, characterised by leaf crown elements surrounding the buccal capsule, consists of *Phascolostrongylus* and *Oesophagostomoides* and occurs exclusively in wombats. Hypodontinea comprises genera with globular buccal capsules, namely *Hypodontus*, *Macropicola* and *Corollostrongylus*. The genera of the Macropostrongyloidinea, including *Macropostrongyloides*, *Paramacropostrongylus*, *Torquenema* and *Wallabicola*, possess cylindrical buccal capsules surrounded by teeth or denticles [2]. The morphological classification of the Phascolostrongylinae has been uncertain due to extensive variation in oral morphology that can be challenging to observe [1, 2]. Therefore, molecular markers, including the first and second region of internal transcribed spacers (ITS-1 and ITS-2) of ribosomal DNA (rDNA), have been utilised for specific identification purposes, detection of genetic variation and phylogenetic analyses within the Phascolostrongylinae [3–9]. In a recent study, two species (*Macropostrongyloides dissimilis* and *Paramacropostrongylus toraliformis*) were found to be divergent from their congeners using ITS markers [9]. Subsequently, morphological examination of these nematodes led to the description of two new genera, namely *Wallabicola dissimilis* (formerly *M. dissimilis*) from the swamp wallaby, *Wallabiocola bicolor* and *Torquenema toraliforme* (formerly *P. toraliformis*) from the eastern grey kangaroo *Macropus giganteus* [10].

Although ITS sequence data can be useful for inferring phylogenetic relationships at the species and genus level, this has not been the case at higher taxonomic levels. Phylogenetic analyses within the superfamily Strongyloidea determined using the ITS-2 marker showed conflicting topologies and low nodal support for the relationships between the Phascolostrongylinae, Oesophagostominae and Chabertiinae [11]. Amino acid sequences derived from the mitochondrial protein-coding genes have been used to validate phylogenetic relationships within *Hypodontus* [12] and *Macropostrongyloides* [13] of the Phascolostrongylinae. These studies validated previous phylogenetic hypotheses based on ITS sequence data with improved nodal support in the phylogenetic analyses.

Therefore, in the present study we assessed the phylogenetic relationships within the subfamily Phascolostrongylinae utilising the amino acid sequence data sets derived from the mitochondrial protein-coding genes. We also assessed the phylogenetic relationship between genera of the Phascolostrongylinae with other subfamilies of the Chabertiidae and with the Strongylidae. Published mitochondrial protein sequences of genera from these families were included in the phylogenetic analyses.

## Methods

### Sample collection and DNA extraction

Adult male and female nematodes used in the current study (Table 1) were obtained from the frozen parasite collection at the School of Veterinary Science, The University of Melbourne. The nematodes were collected from the gastrointestinal tracts of carcasses of hosts from commercial cullings or vehicle collisions (State-issued permits: Victorian Department of Sustainability and Environment 90-053, 93-016, 10000434, 100003649; Queensland Department of Environment and Heritage Protection WA00006125). The samples had been either frozen at − 80 °C or preserved in 70% ethanol and then frozen at − 80 °C as individuals or pools. If preserved in ethanol, worms were rehydrated in distilled water prior to DNA isolation. For morphological identification, the anterior and posterior extremities of each nematode were removed with a scalpel blade, cleared with lactophenol and identified. They were subsequently deposited in the helminthological collection of the South Australia Museum, Adelaide, as voucher specimens (Registration numbers Australian Helminthological Collection [AHC] 36783, 49028, 49037, 49035, 49052, 49051, 49055, 49108). The mid-sections were used for the DNA isolation.

**Table 1:** Species of Phascolostrongylinae included in the current study and reference sequences obtained from GenBank database

Family/subfamily	Species^a^	Host	Site within host^b^	Collection locality^c^	GenBank accession no.	References
Subfamily Phascolostrongylinae	*Oesophagostomoides giltneri* (P)	*Vombatus ursinus*	LI	Flowerdale, Vic, Australia	OK111101	This study
	*Oesophagostomoides longispicularis* (P)	*Vombatus ursinus*	LI	Gippsland, Vic, Australia	OK111102	This study
	*Oesophagostomoides stirtoni* (P)	*Lasiorhinus latifrons*	LI	Swan Reach, SA, Australia	OK111103	This study
	*Phascolostrongylus turleyi* (P)	*Vombatus ursinus*	LI	Flowerdale, Vic, Australia	OK111104	This study
	*Paramacropostrongylus iugalis* (M)	*Macropus giganteus*	S	Miles, Qld, Australia	OK111105	This study
	*Paramacropostrongylus iugalis* (M)	*Macropus giganteus*	S	Charters Towers, Qld, Australia	OK111106	This study
	*Paramacropostrongylus typicus* (M)	*Macropus fuliginosus*	LI	Nyngan, NSW, Australia	OK111107	This study
	*Torquenema toraliforme* (M)	*Macropus giganteus*	LI	Research, Vic, Australia	OK111108	This study
	*Macropostrongyloides mawsonae* (M)	*Macropus giganteus*	LI	Heathcote, Vic, Australia	MW309873	[13]
	*Macropostrongyloides baylisi* (M)	*Osphranter robustus*	LI	Cloncurry, Qld, Australia	MW309874	[13]
	*Macropostrongyloides yamagutii* (M)	*Macropus fuliginosus*	LI	Hattah Lakes, Vic, Australia	MW309875	[13]
	*Macropostrongyloides spearei* (M)	*Osphranter robustus*	LI	Kalgoorlie, WA, Australia	MW309876	[13]
	*Macropostrongyloides phascolomys* (M)	*Vombatus ursinus*	LI	Flowerdale, Vic, Australia	MW309877	[13]
	*Macropostrongyloides woodi* (M)	*Osphranter rufus*	LI	Kalgoorlie, WA, Australia	MW309878	[13]
	*Wallabicola dissimilis* (M)	*Wallabia bicolor*	S	Kamarooka, Vic, Australia	MW309879	[13]
	*Hypodontus macropi* (H)	*Wallabia bicolor*	SI + LI	Hall’s Gap, Vic, Australia	KF361317	[12]
	*Hypodontus macropi* (H)	*Thylogale billardierii*	SI + LI	Launceston, Tas, Australia	KF361318	[12]
	*Hypodontus macropi* (H)	*Macropus robustus*	SI + LI	Barcaldine, Qld, Australia	KF361319	[12]
	*Macropicola ocydromi* (H)	*Macropus fuliginosus*	LI	Waroona, WA, Australia	KF361320	[12]
Subfamily Oesophagostominae	*Oesophagostomum dentatum*	*Sus scrofa domestica*	LI	Chongqing, China	FM161882	[16]
	*Oesophagostomum quadrispinulatum*	*Sus scrofa domestica*	LI	Chongqing, China	FM161883	[16]
	*Oesophagostomum dentatum*	*Sus scrofa domestica*	LI	Werribee, Vic, Australia	GQ888716	[15]
	*Oesophagostomum asperum*	*Capra hircus*	LI	Shaanxi Province, China	KC715826	[17]
	*Oesophagostomum columbianum*	*Ovis aries*	LI	Heilongjiang Province, China	KC715827	[17]
Subfamily Chabertiinae	*Chabertia ovina*	*Ovis aries*	LI	Werribee, Vic, Australia	GQ888721	[15]
	*Chabertia ovina*	*Capra hircus*	LI	Shaanxi Province, China	KF660604	[18]
	*Chabertia ershowi*	*Bos grunniens*	LI	Qinghai Province, China	KF660603	[18]
Family Strongylidae	*Cylicodontophorus bicoronatus*	*Equus caballus*	LI	Heilongjiang Province, China	MH551241	[19]
	*Strongylus vulgaris*	*Equus caballus*	LI	Vic, Australia	GQ888717	[15]
Family Syngamidae	*Syngamus trachea*	*Gymnorhina tibicen*	T	Vic, Australia	GQ888718	[15]

^a^H, Tribe Hypodontinea; M, tribe Macropostrongyloidinea; P, tribe Phascolostrongylinea

^b^LI, Large intestine; S, stomach; SI, small intestine; T, trachea

^c^NSW, New South Wales; Qld , Queensland; SA, South Australia; Tas, Tasmania; Vic, Victoria; WA, Western Australia

Genomic DNA was extracted from individual nematodes using the QiaAmp Micro Kit (Qiagen, Hilden, Germany) following the manufacturer’s protocol for extracting DNA from tissues. For initial molecular identification, the ITS-1 and ITS-2 sequences were determined for each individual using an established PCR-based sequencing method [9]. Prior to sequencing, the quantity and quality of the DNA were determined using the 2200 TapeStation (Agilent Technologies, Santa Clara, CA, USA).

### Sequencing and gene annotation

Illumina TruSeq indexed libraries were prepared using sheared DNA following the manufacturer’s protocol (Illumina Inc., San Diego, CA, USA). Briefly, the steps included: (i) end-repair and A-tailing of the 3′ ends; (ii) ligation of the adaptors; (iii) enrichment of the libraries and purification of the enriched library using Ampure Beads (Beckman Coulter, Brea, CA, USA). The libraries were quantified using the 2200 TapeStation, pooled and sequenced on the Illumina MiSeq platform using the 300 cycle v3 reagent kit (2 × 150 paired-end reads). Illumina library preparation and sequencing were carried out at the Walter and Eliza Hall Institute (WEHI) Genomics Facility, Melbourne, Victoria, Australia.

Raw sequence data in the FASTQ format were filtered for quality in Trimmomatic v.0.38 [14] prior to de novo assembly employing the program Spades v3.13.0 under default parameters. For each assembly, the 12 protein-coding genes of the mitochondrial genome were identified by local sequence alignment (6 reading frames) using the amino acid sequence inferred from corresponding genes of reference mitochondrial genomes, using an established workflow system [15]. The reference mitochondrial genomes used were selected based on sequence similarities using Basic Local Alignment Search Tool (BLAST) for sequence analysis [20]. The mitochondrial genome sequence of *Hypodontus macropi* (NC023083) was used as a reference for *Paramacropostrongylus*, *Torquenema* and *Wallabicola*, and that of *Oesophagostomum dentatum* (NC013817) was used as the reference for *Oesophagostomoides* and *Phascolostrongylus*. The nucleotide sequences of the 12 protein-coding genes of each species included in this study were deposited in the GenBank database under the accession numbers OK111101–OK111108.

### Sequence comparison and phylogenetic analyses

The nucleotide and amino acid sequences of the 12 mitochondrial protein-coding genes were aligned separately using CLUSTAL W [21] and MUSCLE [22] followed by a concatenation of the alignments using MEGA software version X [23]. The alignment included previously published mitochondrial protein-coding gene sequences of species of Phascolostrongylinae, Chabertiinae, Oesophagostominae, Strongylidae and Syngamidae (Table 1). Pairwise comparisons were calculated for the nucleotide and amino acid sequences using MEGA software. The nucleotide diversity across each of the protein-coding genes was determined using sliding window analyses (SWAN) carried out in the DnaSP v.5 program [24] using a sliding window of 100 bp and 25-bp steps.

Phylogenetic analysis was conducted using concatenated and aligned amino acid sequences derived from all 12 mitochondrial protein-coding genes. The phylogenetic analysis was conducted using Bayesian inference (BI) in MrBayes v.3.2.7 [25]. The optimal partitioning schemes and substitution model for the BI analyses were determined using PartitionFinder 2 [26] for amino acids, with a model selection set to the Akaike information criterion and greedy search algorithm. According to PartitionFinder, the amino acid sequence alignment was partitioned into eight subsets consisting of subset 1 (cytochrome* c* oxidase [*cox*] subunit 1-2), subset 2 (*cox*3, nicotinamide adenine dinucleotide hydrogen dehydrogenase [*nad*] subunit 4), subset 3 (*nad*5), subset 4 (*nad*6, *nad*2), subset 5 (*nad*4L, *nad*1), subset 6 (adenosine triphosphate synthase subunit 6 [*atp*6]), subset 7 (cytochrome* b* [*cob*]) subset 8 (*cox*3). The Mtmam (Mitochondrial Mammalia) model was the evolutionary model for all subsets except subset 6 (general reversible Markov model for amino acid substitution of mitochondrial proteins [Mtrev] model). The BI analysis was conducted with four chains each of 10 million Markov chain Monte Carlo iterations, sampling every 1000th generation for four independent runs. Convergence was determined by the average standard deviation of split frequencies of > 0.01, with the potential scale reduction factor approaching 1. The first 25% of the sampled trees were discarded as burnin and the Bayesian consensus tree was constructed from the remaining trees. The BI trees were visualised in FigTree v.1.4.4 [27]. *Syngamus trachea* (GQ888718) belonging to the family Syngamidae was used as the outgroup.

## Results

### Nucleotide and amino acid sequence comparisons

**Fig. 1:**
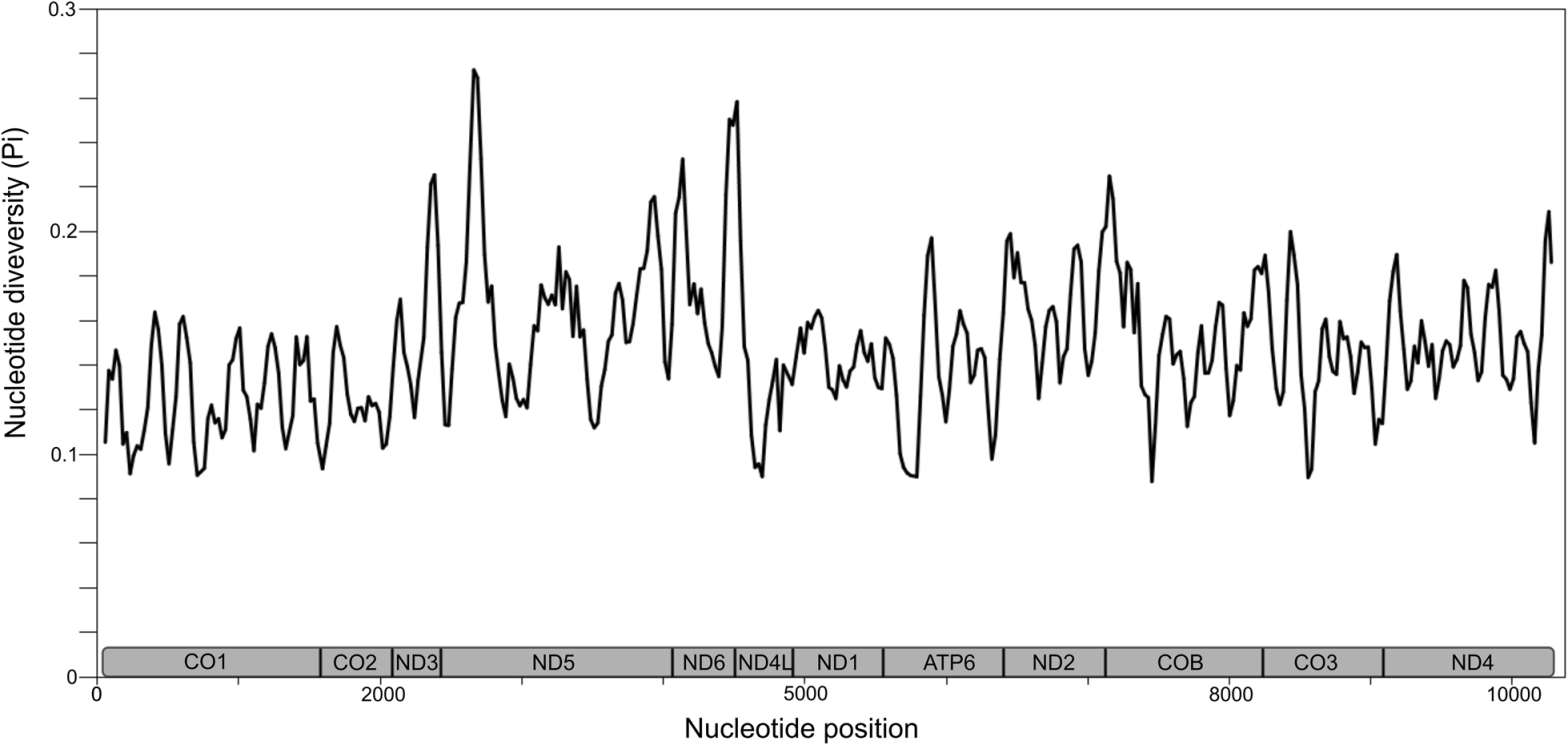
Nucleotide diversity (Pi) across 12 concatenated mitochondrial protein-coding genes (*y*-axis) of eight genera of Phascolostrongylinae, two genera of Strongylidae and one genus each of Oesophagostominae, Chabertiinae and Syngamidae. Nucleotide diversity was calculated in DnaSP version 6 software using a window of 100 bp and 25-bp steps. The nucleotide position (base pairs) is indicated on the *x*-axis next to the boundaries between mitochondrial protein-coding genes. CO1 – 3 = Cytochrome c oxidase subunit 1 – 3; ND1 – 6, 4L = Nicotinamide adenine dinucleotide hydrogen dehydrogenase subunits 1 – 6 and 4L; ATP6 = Adenosine triphosphate synthase subunit 6; COB = Cytochrome B

The SWAN revealed that the nucleotide diversity across the alignment of 12 concatenated mitochondrial protein-coding genes ranged from 0.084 to 0.290 (Fig. 1). The 5′ prime end of the *nad*5 gene exhibited the highest level of nucleotide diversity, whereas *cox*1 was the most conserved gene.

Pairwise amino acid sequence differences among species of Phascolostrongylinae ranged between 0.60% (between two specimens of *Paramacropostrongylus iugalis*) and 10.6% (between *Phascolostrongylus turleyi* and *Macropostrongyloides baylisi*) (Table 2). The genus *Wallabicola* was most similar to *Paramacropostrongylus typicus*, with 5.9% sequence variation, compared to 8.6% variation from *Macropostrongyloides* in which it was formerly placed. The amino acid sequence of *Torquenema* was most similar to that of *Paramacropostrongylus typicus* (6.5% sequence variation), the genus to which it was formerly assigned. Amino acid sequence variation between the wombat-specific genera *Phascolostrongylus* and *Oesophagostomoides* spp. was small (4.7–5.0%), with *Ph. turleyi* and *Oesophagostomoides longspicularis* sharing the greatest sequence similarities. Interestingly, the amino acid sequences of *Phascolostrongylus* and *Oesophagostomoides* spp. shared greater similarities with two species from the subfamily Oesophagostominae, *Oe. dentatum* and *Oesophagostomum quadrispinulatum* (6.8–7.3% sequence variation) compared to genera from the same subfamily (9.1–10.6% sequence variation) (Table 2).

**Table 2:** Pairwise difference (%) of the nucleotide (top) and amino acid (bottom) sequences derived from the 12 concatenated mitochondrial protein-coding genes among species of the Phascolostrongylinae, Oesophagostominae and Chabertiinae

Species	1	2	3	4	5	6	7	8	9	10	11	12	13	14	15	
1 *P. iugalis* (OK111105)	–	13.50	13.57	14.97	14.04	14.64	14.92	14.69	14.78	15.16	14.57	14.30	15.45	15.61		15.90
2* T. toraliforme* (OK111108)	6.90	–	13.80	14.47	13.97	13.76	15.71	15.48	14.95	15.41	14.95	14.67	15.95	15.74		16.14
3* W. dissimilis* (MW309879)	6.04	6.73	–	15.35	15.02	14.60	15.58	15.83	15.53	16.04	15.23	15.18	16.17	16.02		16.27
4* M. baylisi* (MW309874))	8.50	8.12	8.68	–	15.32	15.13	16.36	16.60	16.50	16.29	15.98	15.58	16.50	16.27		16.97
5 *H. macropi* (KF361319)	6.73	6.49	6.49	7.94	–	13.73	15.80	16.17	15.58	16.05	15.22	15.74	16.28	15.83		16.24
6* Ma. ocydromi* (KF361320)	7.41	7.14	7.20	7.94	6.07	–	15.54	15.94	15.62	16.28	15.63	16.08	16.16	15.63		16.34
7* O. giltneri* (OK111101)	8.68	8.98	8.56	10.22	9.33	9.57	–	12.68	12.78	12.61	14.80	14.87	15.94	15.39		16.41
8 *O. longispicularis* (OK111102)	8.27	8.44	8.44	10.22	9.07	9.63	4.89	–	12.68	12.68	14.81	14.69	15.84	15.30		16.47
9* O. stirtoni* (OK111103)	8.24	8.39	8.47	10.22	8.86	9.39	4.95	5.01	–	12.75	14.58	14.45	15.56	14.90		15.72
10* Ph. turleyi* (OK111104)	9.07	9.24	8.95	10.64	9.45	9.96	5.01	4.72	4.74	–	14.72	15.04	16.12	15.64		16.30
11 *Oe. dentatum* (GQ888716)	8.50	8.74	8.36	10.22	8.15	9.33	7.08	6.90	6.76	7.32	–	12.10	15.39	4.79		15.85
12* Oe.quadrispinulatum* (FM161883)	8.09	8.71	8.27	10.10	8.36	9.33	7.29	6.99	6.96	7.41	3.44	–	14.63	14.51		15.69
13* Oe. asperum* (KC715826)	11.02	11.17	10.61	12.65	11.23	11.59	10.19	10.43	10.43	11.17	10.04	9.39	–	15.07		15.37
14 *Oe. columbianum* (KC715827)	11.05	11.26	10.73	12.27	11.14	11.32	10.25	10.13	10.04	10.34	8.98	8.80	11.85	–		15.48
15 *C. ovina* (GQ888721)	11.47	11.59	11.23	13.07	11.38	11.91	10.73	11.02	10.49	11.35	9.87	9.90	11.69	11.50		–

### Phylogenetic analyses

**Fig. 2:**
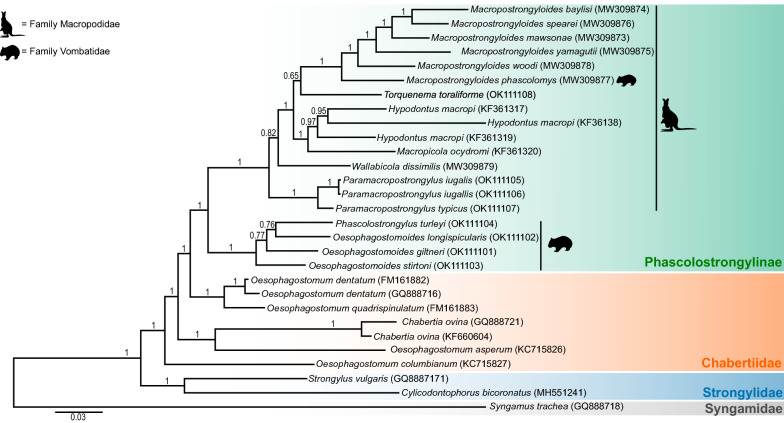
Topology of the Bayesian inference phylogenetic analyses inferred from the concatenated alignment of 12 mitochondrial protein-coding genes of the genera of Phascolostrongylinae, Oesophagostominae, Chabertiinae (Chabertiidae) and Strongylidae. Nodal support is indicated as posterior probabilities of the Bayesian inference analysis. *Syngamus trachea* from the family Syngamidae was used as the outgroup. The host families (Macropodidae or Vombatidae) in which the species of Phascolostrongylinae occur are represented by icons. The scale bar indicates the number of inferred substitutions per amino acid site

The BI analysis showed strong support for the clustering of the genera of Phascolostrongylinae (Fig. 2). The tree topology showed that the genera of the Phascolostrongylinae distributed between two clades, with the larger clade comprising members of the tribes Macropostrongylinea and Hypodontinea from macropodid marsupials and the smaller clade comprising the tribe Phascolostrongylinea from vombatid marsupials (Fig. 2). The tribe Phascolostrongylinea comprising the wombat-specific genera *Phascolostrongylus* and *Oesophagostomoides* formed a strongly supported monophyletic assemblage (posterior probability [pp] = 1), sister to the species occurring in macropodid marsupials and *Macropostrongyloides phascolomys* from the common wombat. The clade comprising *Hypodontus* and *Macropicola* belonging to the tribe Hypodontinea was monophyletic, with strong nodal support (pp = 1). The tribe Macropostrongyloidinea was paraphyletic. *Macropostrongyloides* was placed as a sister group to the tribe Hypodontinea, and *Wallabicola dissimilis* from the stomach of the swamp wallaby was placed on a branch external to the clade comprising *Hypodontus* and *Macropostrongyloides*. The position of *T. toraliforme*, sister to *Macropostrongyloides* lacked nodal support (pp = 0.65). *Paramacropostrongylus typicus* and *P. iugalis* from grey kangaroos grouped together with strong nodal support (pp = 1).

Within the tribe Oesophagostominea, *Oesophagostomum dentatum* and *Oesophagostomum quadrispinulatum* formed a strongly supported (pp = 1) clade sister to the subfamily Phascolostrongylinae, with the exclusion of *Oe*. *asperum* and *Oe. columbianum. Oesophagostomum asperum* clustered with *Chabertia ovina* and *C. erschowi* (tribe Chabertiinea) whilst *Oe. columbianum* was placed on an external branch. The genera *Strongylus vulgaris* and *Cyclicodontophorus bicoronatu*s are representative of the family Strongylidae that is sister to the subfamilies Phascolostrongylinae, Oesophagostominae and Chabertiinae, all of which belong to the family Chabertiidae. Overall there were six genera comprising at least two species included in the study, four of these were monophyletic (*Macropostrongyloides*, *Hypodontus*, *Paramacropostrongylus *and *Chabertia*) and two were paraphyletic (*Oesophagostomoides *and *Oesophagostomum*).

## Discussion

The current study utilised the amino acid sequences derived from the mitochondrial protein-coding genes to assess the phylogenetic relationships of the subfamily Phascolostrongylinae. The tree topology showed that the nine genera currently placed in the subfamily Phascolostrongylinae clustered together with strong nodal support. Overall, the use of mitochondrial amino acid sequence data sets resulted in well-supported relationships among most but not all taxa included in the phylogenetic analyses. There was good support for the monophyly of the morphologically defined tribes Phascolostrongylinea and Hypodontinea but not Macropostrongyloidinea, consistent with the findings from a previous study using ITS sequence data sets [9].

Contrary to the previous phylogeny based on ITS sequences [9], the BI analysis in the current study showed strong nodal support for the clade containing the genera *Macropicola* and *Hypodontus.* This relationship corresponded with the tribe Hypodontinea erected by Beveridge [2]. The classification of *Hypodontus* and *Macropicola* has been debated in the past as their morphology shares little resemblance to that of other strongyloid nematodes infecting marsupials. *Hypodontus* was initially placed in the family Ancylostomatidae as it resembled hookworms, due to its ventrally bent anterior extremity and the presence of two cutting plates within the buccal capsule [28]. Subsequently, Inglis et al. [29] transferred *Hypodontus* to the subfamily Globocephaloidinae within the Trichostrongyloidea without a clear explanation. However, it was moved back to the Strongyloidea and reassigned as a hookworm in the subfamily Uncinariinae by Durette-Desset et al. [30], having formerly been placed in the hookworm subfamily Bunostomatinae by Skryabin et al. [31]. The genus *Macropicola*, also in the tribe Hypodontinea, was initially classified as a hookworm in the subfamily Globocephalinae based on its globular buccal capsule and three oesophageal teeth [32]. However, Lichtenfels [33] argued that both *Hypodontus* and *Macropicola* belonged to the subfamily Strongylinae in the Strongylidae based on the globular buccal capsule, oesophageal teeth, the Y-shaped female ovejector and a pre-anal vulva. Finally, in the most recent revision, Beveridge [2] assigned *Hypodontus* and *Macropicola* to the tribe Hypodontinea, along with *Corollostrongylus* within the subfamily Phascolostrongylinae, based on their large globular or subglobular buccal capsules that are either straight or bent dorsally or ventrally. This morphological grouping was supported by the mitochondrial protein sequence data in the current study and in a previous study on hookworms which showed the exclusion of *Hypodontus* and *Macropicola* from the Strongylinae and Ancylostomatoidea [34]. Although *Hypodontus* closely resembles hookworms, molecular data suggest that their morphological resemblances could be a result of convergent evolution. Three genotypes of *Hypodontus macropi*, each from a different host (*Osphranter robustus*, *Thylogale billardierii* and *Wallabia bicolor*) were included in the analyses. The tree topology showed that the specimens of *Hypodontus* from each host are distinct, with the specimen from the Tasmanian pademelon (*T. billardierii*) being the most divergent. This finding is consistent with a previous phylogenetic analysis also based on the mitochondrial protein sequence data [12], in addition to ITS-based studies [3, 6]. In the absence of consistent morphological differences between the distinct genotypes, *H. macropi* remains a cryptic species complex.

Analyses of both the current mitochondrial and previous ITS data sets placed *Torquenema* external to the clade comprising *Macropostrongyloides*, but with low branch support. Although *Torquenema* can be clearly distinguished from *Macropostrongyloides* by its occurrence in the stomach of its host and its prominent cervical collar, they share some common features, including a Y-shaped ovejector and small peri-oral denticles [10, 35]. Mitochondrial and ITS data support the separation of *Torquenema* from its previous position within *Paramacropostrongylus*. However, its current phylogenetic position close to *Macropostrongyloides* is uncertain due to the lack of nodal support.

The current phylogenetic analyses placed *W. dissimilis* (formerly *Macropostrongyloides dissimilis*) external to the clade comprising the genus *Macropostrongyloides* and the tribe Hypodontinea. This finding differs from that of a previous study using ITS sequence data which showed a strongly supported grouping of *W. dissimilis* and *Paramacropostrongylus* spp., both occurring within the stomach of macropodid hosts [9]. However, the current topology based on mitochondrial protein sequence data lacks nodal support (pp = 0.82). Therefore, additional data, including a larger number of representatives of this species and potentially a different genetic marker, are required to validate the phylogenetic position of *Wallabicola*.

The current and previous study based on ITS sequence data showed strong support for the monophyletic grouping of the genera within tribe Phascolostrongylinea from vombatid marsupials. The topologies of the current and previous phylogenetic analyses based on ITS sequence data showed the grouping of *Ph. turleyi* and *O. longispicularis* with *O. stirtoni* on a sister branch [9]. However, nodal support was higher in the ITS study [9]. Although *Oesophagostomoides* appears to be paraphyletic due to the position of *Phascolostrongylus*, there are no morphological features to support this apparent paraphyly. These two genera are considered valid and clearly distinguishable morphologically. The buccal capsules of *Phascolostrongylus* are shallower with thicker walls compared to *Oesophagostomoides* and have a greater number of external leaf crown elements [36]. *Oesophagostomoides longispicularis* and *Ph. turleyi* often occur at a high prevalence and together within the same host, whereas *O. giltneri* has been less commonly encountered in the common wombat [36].

The phylogenetic analysis showed strong support for the sister relationship between the genera of the subfamily Phascolostrongylinae with *Oe. dentatum* and *Oe. quadrispinulatum* (subfamily Oesophagostominae) which was not evident in a previous study based on ITS-2 sequence data [11]. The genus *Oesophagostomum* occurs in the caecum of ungulates, rodents and primates [37]. There are several subgenera within *Oesophagostomum,* some of which corresponded to those included in the current phylogenetic analyses. The separation of *Oe. asperum* from *Oe. columbianum* in the phylogenetic tree corresponded with their different subgenera, *Hysteracrum* and *Proteracrum*, respectively, whereas the clade closest to the subfamily Phascolostrongylinae, comprising *Oe. dentatum* and *Oe. quadrispinulatum*, both belong to the subgenus *Oesophagostomum*. The genus *Chabertia* appears to be monophyletic in the current analyses, although *C. ovina* from a sheep in Australia (GQ888271) was slightly divergent from the one from a goat from China (KF660603), suggesting intraspecific genetic variation within this species. The position of *Chabertia*, nested among the Oesophagostominae, is consistent with the topology of a previous phylogenetic tree based on the analysis of ITS-2 sequence data [38], suggesting that the current classifications of the Chabertiinae and Oesophagostominae may require further investigation.

The genera *Oesophagostomoides* and *Oesophagostomum* were considered synonyms by Popova [39] and Yamaguti [40] based on their morphological similarities. The key differential feature between *Oesophagostomoides* and *Oesophagostomum* are the Y-shaped ovejectors in the former genus compared with J-shaped ovejectors in the latter and the shape of their cervical papillae, which are bipartite in the former but conical in the latter [36]. The sister relationship between *Oesophagostomum* and subfamily Phascolostrongylinae found in the current study raises the question of whether the Oesophagostominae could have been the predecessors of the Strongyloidea in marsupials. However, this proposal was deemed improbable, since the most likely host, rodents, arrived in Australia after the expansive radiation of the marsupials which was believed to coincide with the radiation of their parasitic nematodes [41]. However, in a study using* 18S* nuclear ribosomal gene sequence data [42], *Cyclodontostomum purvursi* from Australian rodents grouped closely to *Chabertia ovina*. Further speculation on the association between the Oesophagostominae and Phascolostrongylinea is beyond the scope of this study and would require additional mitochondrial sequence datasets of other *Oesophagostomum* spp. and *Cyclodontostomum*, both of which are currently unavailable.

Although the current phylogenetic analyses suggest the monophyly of the Phascolostrongylinae, not all species were included. These species were *Corollostrongylus hypsiprymnodontis* (tribe Hypodontinea) from the musky-rat kangaroo *Hypsiprymnodon moschatus* [43], *Macropostrongyloides dendrolagi* (tribe Macropostrongyloidinea) from tree kangaroos, *Dendrolagus* spp. [44], *Macropostrongyloides lasiorhini* (tribe Macropostrongyloidinea) from the southern hairy-nosed wombat *Lasiorhinus latifrons* and *Oesophagostomoides eppingensis* (tribe Phascolostrongylinea) from the northern hairy-nosed wombat *Lasiorhinus krefftii* [45]. Each of these species occurs in rare or endangered hosts and, therefore, specimens for molecular analyses were not readily available through opportunistic sampling.

Overall, the current study demonstrated that phylogenetic inference based on amino acid sequences of mitochondrial protein-coding genes generated strong nodal support. However, the conflicting evidence between the mitochondrial- and ITS-based analyses in the case of *Wallabicola* highlights the importance of using multiple genetic markers to independently validate phylogenetic hypotheses. Furthermore, multiple samples from the same host populations should ideally be included in such analyses. However, this can be challenging due to limitations in collecting sufficient samples from wildlife hosts. Sliding window analyses of the mitochondrial protein-coding genes in current and previous studies suggest that there are variable regions flanked by conserved regions in genes such as *nad*1 and *nad*5. PCR-coupled sequencing of such potentially phylogenetically informative regions could reduce the cost and amount of DNA required compared to sequencing mitochondrial genomes.

## Conclusions

Utilising the amino acid sequences translated from the mitochondrial protein-coding genes, this study provided evidence for the grouping of the currently recognised genera of the Phascolostrongylinae. Findings from the current study contribute to our understanding of the phylogenetic position of the subfamily Phascolostrongylinae within the Chabertiidae. This study suggests that the amino acid sequence data could be used to assess the relationship within the subfamily Cloacininae which has yet to be resolved [46]. The inclusion of genera of the subfamily Cloacininae from macropodid marsupials in future studies could further elucidate the phylogenetic relationships among the strongyloid nematodes from Australian marsupials.

## Data Availability

All data generated or analysed during this study are included in this article. The sequence data generated in this study are available in the NCBI database under accession numbers OK111101–OK111108. Molecular vouchers specimens have been deposited in the South Australian Museum under the registration numbers AHC 36783, 49028, 49037, 49035, 49,052, 49051, 49055, 49108.

## Abbreviations

AHC: Australian Helminthological Collection
atp6: Adenosine triphosphate synthase subunit 6
BI: Bayesian inference
BLAST: Basic Local Alignment Search Tool
cob: Cytochrome* b*
cox1-3: Cytochrome* c* oxidase subunit 1-3
ITS-1: Internal transcribed spacer region 1
ITS-2: Internal transcribed spacer region 2
Mtmam: Mitochondrial Mammalia
Mtrev: General reversible Markov model for amino acid substitution of mitochondrial proteins
nad1–6, 4L: Nicotinamide adenine dinucleotide hydrogen dehydrogenase subunits 1–6 and 4L
MUSCLE: Multiple Sequence Comparison by Log-Expectation program
pp: Posterior probability
rDNA: Ribosomal DNA
SWAN: Sliding window analyses
WEHI: Walter and Eliza Hall Institute
